# Deciphering the potential of a plant growth promoting endophyte *Rhizobium* sp. WYJ-E13, and functional annotation of the genes involved in the metabolic pathway

**DOI:** 10.3389/fmicb.2022.1035167

**Published:** 2022-11-03

**Authors:** Xiaoping Huang, Zhanghui Zeng, Zhehao Chen, Xiaxiu Tong, Jie Jiang, Chenjing He, Taihe Xiang

**Affiliations:** ^1^College of Life and Environmental Sciences, Hangzhou Normal University, Hangzhou, China; ^2^Zhejiang Provincial Key Laboratory for Genetic Improvement and Quality Control of Medicinal Plants, Hangzhou, China

**Keywords:** *Curcuma wenyujin*, nitrogen metabolism, *Rhizobium* sp., genome, plant growth-promoting rhizobacteria

## Abstract

Plant growth-promoting rhizobacteria (PGPR) are well-acknowledged root endophytic bacteria used for plant growth promotion. However, which metabolites produced by PGPR could promote plant growth remains unclear. Additionally, which genes are responsible for plant growth-promoting traits is also not elucidated. Thus, as comprehensive understanding of the mechanism of endophyte in growth promotion is limited, this study aimed to determine the metabolites and genes involved in plant growth-promotion. We isolated an endophytic *Rhizobium* sp. WYJ-E13 strain from the roots of *Curcuma wenyujin* Y.H. Chen *et* C. Ling, a perennial herb and medicinal plant. The tissue culture experiment showed its plant growth-promoting ability. The bacterium colonization in the root was confirmed by scanning electron microscopy and paraffin sectioning. Furthermore, it was noted that the WYJ-E13 strain produced cytokinin, anthranilic acid, and L-phenylalanine by metabolome analysis. Whole-genome analysis of the strain showed that it consists of a circular chromosome of 4,350,227 bp with an overall GC content of 60.34%, of a 2,149,667 bp plasmid1 with 59.86% GC, and of a 406,180 bp plasmid2 with 58.05% GC. Genome annotation identified 4,349 putative protein-coding genes, 51 tRNAs, and 9 rRNAs. The CDSs number allocated to the Kyoto Encyclopedia of Genes and Genomes, Gene Ontology, and Clusters of Orthologous Genes databases were 2027, 3,175 and 3,849, respectively. Comparative genome analysis displayed that *Rhizobium* sp. WYJ-E13 possesses the collinear region among three species: *Rhizobium acidisoli* FH23, *Rhizobium gallicum* R602 and *Rhizobium phaseoli* R650. We recognized a total set of genes that are possibly related to plant growth promotion, including genes involved in nitrogen metabolism (*nifU*, *gltA*, *gltB*, *gltD*, *glnA*, *glnD*), hormone production (*trp ABCDEFS*), sulfur metabolism (*cysD*, *cysE*, *cysK*, *cysN*), phosphate metabolism (*pstA*, *pstC*, *phoB*, *phoH*, *phoU*), and root colonization. Collectively, these findings revealed the roles of WYJ-E13 strain in plant growth-promotion. To the best of our knowledge, this was the first study using whole-genome sequencing for *Rhizobium* sp. WYJ-E13 associated with *C. wenyujin*. WYJ-E13 strain has a high potential to be used as *Curcuma* biofertilizer for sustainable agriculture.

## Introduction

Endophytes are an endosymbiotic group of microorganisms ubiquitous in nature and well-known to dwell inside plants without causing any apparent harmful effect on the host plant (Kaul et al., 2016; Dubey et al., 2020). Among plant microbiota, endophytes can be found in most plant species, and have been recovered from various tissues, including roots, leaves, stems, flowers, fruits, and seeds (Elmagzob et al., 2019). With the rapid development of science, plant growth-promoting rhizobacteria (PGPR) have attracted more attentions due to their plant growth and development promoting effects.

Certainly, the beneficial relationships conferred by endophytes to host plants are worth revealing, which could be displayed from three different aspects, including growth promotion of their host plants, the secretion of bioactive compounds to increase the stress resistance of the host plants, and the accumulation of important secondary metabolites produced by endophytes (Jia et al., 2016). Particularly, mounting research has paid attentions to the effects of endophytes on plant growth. For example, the application of endophytic fungus *Paecilomyces variotii* has resulted in improved growth in the development of pepper seedlings (Moreno-Gavíra et al., 2020). *Enterobacter roggenkampii* ED5 and *Pseudomonas aeruginosa* B18 have been both able to enhance the growth of sugarcane (Guo et al., 2020; Singh, P., et al., 2021). *Bacillus velezensis* LDO2 has been able to increase the root length of peanut plants (Chen et al., 2019). Two strains *Microbacterium* spp. and *Streptomyces* spp. have significantly promoted ice plant growth compared with the control (Kataoka et al., 2021). Generalist endophyte *Phomopsis liquidambaris* colonization of *Oryza sativa* L. has promoted plant growth under nitrogen starvation (Zhou et al., 2022). Notably, the effect of plant growth promotion depends on phosphate solubilization (Liu et al., 2014; Oteino et al., 2015), nitrogen fixation (Guo et al., 2020), hormone indole-3-acetic acid (IAA) synthesis (Hassan, 2017; Harman et al., 2021), and augmenting the resistance of plants to insects and pathogens by producing bioactive metabolites (Etminani and Harighi, 2018). Besides, beneficial endophytes have also been found in many genera, including *Stenotrophomonas* sp. (Hazarika et al., 2021), *Pantoea cypripedii* and *Kosakonia arachidis* (Singh, R. K., et al., 2021), *Pennisetum giganteum* (Yankey et al., 2022), and *Klebsiella aerogenes* (Cheng et al., 2022).

*Curcuma wenyujin* Y.H. Chen *et* C. Ling is a perennial herb and medicinal plant belonging to the Zingiberaceae family, which is mainly distributed in Wenzhou area of Zhejiang province, China. Its raw rhizomes, steamed rhizomes, and steamed roots constitute three herbal medicines currently listed in the Chinese Pharmacopoeia: pian-jiang-huang, wen-e-zhu and wen-yu-jin, respectively (Li et al., 2021). Accordingly, the research on *C. wenyujin* has been focused on its various anticancer activities in human renal cell carcinoma (Liu et al., 2019), lung cancer (Wu P. et al., 2021), and hepatocellular carcinoma (Wu et al., 2022). Nevertheless, the interaction between endophytes and *Curcuma* plant has also been gradually explored. For example, the endophyte *P. aeruginosa* (BacDOB-E19) have shown multiple plant growth promoting traits, such as increasing plant height and fresh rhizome yield/plant in comparison with untreated control under greenhouse conditions (Vinayarani and Prakash, 2018). Quinalphos-tolerant endophytic *Bacillus* sp. Fcl1 has been isolated from the rhizome of *Curcuma longa*, and shown its toxicity-alleviating effect in *Vigna unguiculata* (Juby et al., 2021). Two species of endophytic *Paenibacillus* have been identified from the rhizome as indole 3 acetic acid producers, thus have potential growth-regulating effect in rhizomes (Aswathy et al., 2013). However, no plant growth-promoting endophytic rhizobium isolated from *C. wenyujin* has been reported so far.

The Rhizobiaceae family covers a series of different bacterial genera, including *Rhizobium*, *Sinorhizobium*, *Bradyrhizobium*, *Azorhizobium*, and *Allorhizobium* (Manasa et al., 2017). The beneficial symbiotic relationships between the *Rhizobium* and leguminous crops have been first studied in stem and root nodulating bacteria (Frank, 1889). Generally, most of the rhizobial species are endophytes and colonize intracellularly for root growth promotion by both direct and indirect approaches (Mus et al., 2016). Rhizobia is also employed as PGPR to increase plant growth through the production of hormones, nitrogen fixation, phosphate solubilization, and siderophore development (Pravin et al., 2016). Furthermore, rhizobium is well-known for plant microbiome due to its growth-promoting attributes (Ferreira et al., 2019). Among all rhizobial species, *Bradyrhizobium japonicum* is suitable for siderophore production, and *Rhizobium leguminosarum trifolii* is suitable for phosphate solubilization and IAA production (Garcia-Fraile et al., 2012). Hence, the isolation, screening, and exploitation of efficient endophytic bacteria in agricultural practices have great significance.

Which metabolites produced by endophytic rhizobia could promote plant growth remains unknown. Moreover, an *in-silico* study of various strains has revealed the types of genes and their number that are responsible for the plant growth-promoting traits (Moszer, 1998). Thus, a complete genome study can be used to categorize genes implicated in the positive effects of PGPR, aiding the clarification of plant growth-promoting molecular and functional mechanism (Qin et al., 2017). Therefore, a complete genome sequence and metabolite profiling accessibility of the endophyte will provide an opportunity to understand the mechanism of plant-growth promotion.

This study aimed to (i) isolate and identify the *Rhizobium* strains from the root of *C. wenyujin*, (ii) explore their plant growth-promoting ability, (iii) examine the colonization pattern of WYJ-E13 strain by scanning electron microscopy (SEM) and paraffin sectioning, (iv) perform metabolome analysis, and (v) to sequence the WYJ-E13 genome. To the best of our knowledge, this is the first study of the metabolome and whole-genome analysis of endophyte *Rhizobium* isolated from the *C. wenyujin* root as a potential biofertilizer to improve plant growth.

## Materials and methods

### Materials and sample collection

The seedlings of *C. wenyujin* Y.H. Chen *et* C. Ling were provided by Zhejiang Wenzhou Tianhe Biotechnology Co., LTD. The seedlings were planted in the climate chamber of Hangzhou Normal University (longitude: 120° 02′ E, latitude: 30° 29′ N), Zhejiang province, China. After growth of 5 months, the tuberous root with a diameter of 2 cm was sampled for further isolation of endophytes.

Furthermore, the plantlets of *C. wenyujin* Y.H. Chen *et* C. Ling were also cultivated and obtained *via* tissue culture in our laboratory.

### Isolation and purification of the plant endophytes

The tuberous root sample was rinsed with tap water, soaked in 75% ethanol for 1 min, rinsed with sterilized double-distilled water, disinfected in 0.1% HgCl_2_ for 15 min, and rinsed three times with sterile water. Subsequently, the sample was cut into small pieces of 5 mm of length, and placed onto the potato dextrose agar (PDA) medium containing 50 mg/l streptomycin with the cutting surface toward the medium. Each petri dish of the medium had four root segments of the tuberous root. Then, the root segments were cultured at 28°C for 5 days. Different colonies were selected and streaked on PDA medium to check the purity. The last rinse water was spread onto the medium and also placed at 28°C in an incubator to check the disinfection method accomplishment.

### Morphological and molecular identification of the isolated endophytes

Morphological identification of endophytic bacteria was performed based on colony’s surface morphology, edge shape, and color. Molecular characterization was performed by 16S rDNA sequencing according to our previous study (Song et al., 2017). In detail, bacteria genomic DNA was extracted using the Ezup column bacteria genomic DNA purification Kit (Sangon Biotechnology Co., Ltd., Shanghai, China). The universal primers 27f (5′-AGAGTTTGATCMTGGCTCAG-3′) and 1492r (5′-TACGGYTACCTTGTTACGACTT-3′) were designed and synthesized. Next, Polymerase chain reaction (PCR) was performed, and the PCR products were recycled, purified, and ligated to the pMD19-T vector. The ligation products were transformed into *E. coli* DH5α cells. Positive clones were selected, and insertions in plasmid were sequenced. The sequences were analyzed by NCBI BLAST online software to determine the species of isolated endophytic bacteria. Phylogenetic tree was performed using MEGA X software.

### Inoculation of *Rhizobium* sp. strain WYJ-E13 to the plantlets

Prior to inoculation on plants, the bacterium solution of *Rhizobium* sp. strain WYJ-E13 was prepared. First, WYJ-E13 was added into 100 ml PDA liquid medium and grown at 150 rpm for 12 h at 28°C in constant temperature shaker. After centrifugation at 12000 rpm for 15 min, the obtained sediment was diluted with fresh MS medium to an OD_600_ value of about 0.02, and the resuspend bacterium solution was obtained. Next, the plantlets of *C. wenyujin* Y.H. Chen *et* C. Ling were inoculated on MS solid medium containing 2 mg/l 6-BA and 1 mg/l indoleacetic acid (IAA). Then, 10 μl WYJ-E13 bacterium solution was added onto the base part of the plantlets. Lastly, the plantlets were grown at 22 ± 2°C for 2000 lux with 12 h light condition in a constant temperature incubator. After growth of 15 and 35 days, the growth traits of plantlets were observed.

### Root colonization of *Rhizobium* sp. strain WYJ-E13

The root colonization inside the *C. wenyujin* Y.H. Chen *et* C. Ling was confirmed by scanning electron microscopy (SEM) techniques. For SEM analysis, the root tissues were cut into small pieces and fixed in glutaraldehyde solution overnight at 4°C. The samples were washed three times with double-distilled water and dehydrated in 30, 50, 70, 90, 95 and 100% ethanol for 15 min and then isoamyl acetate for 15 min. After drying the samples with a critical point dryer, the colonization of *Rhizobium* sp. strain WYJ-E13 was observed by SEM.

For paraffin sectioning analysis, the root and bud tissues were fixed in FAA solution for 24 h. Subsequently, the root and bud tissues were washed with 70% ethanol and dehydrated by passing along a graded ethanol series. Xylene was used as a transitional fluid prior to paraffin wax infiltration and embedding. Transverse sections were obtained using LEICA CM 3050 cryostat, and root sections were 8 μm thick. Double staining (1% safranin for 10 min and 1% fast green for 20 s) was used for the preparation of permanent slides. Finally, the images were captured by Olympus CX22LED light microscope.

### Extraction of metabolites produced by *Rhizobium* sp. strain WYJ-E13

The metabolites produced by *Rhizobium* sp. strain WYJ-E13, named as WYJ-FB, were extracted. First, the sample was freeze-dried and vortexed in 1 ml cold mixture containing 40% acetonitrile, 40% methanol, and 20% water. Subsequently, the sample was sonicated for 1 h in ice bath and incubated at −20°C for 1 h to precipitate protein. After centrifugation at 16000 rpm for 20 min at 4°C, the obtained supernatant was dried in a high-speed vacuum desiccator. The sample was mixed with 100 μl solution of acetonitrile: water (1:1) for 1 min to end derivatization. After centrifugation, 5 μl supernatant was analyzed through liquid chromatography electrospray ionization tandem mass spectrometry. Additionally, the sterilized LB medium of the same batch was set as the control, named as LB.

### Chromatographic separation of metabolites

Chromatographic separation was performed on an ultra-high performance liquid chromatography system (Agilent 1,290 Infinity, Waldbronn, Germany) equipped with HILIC column. The column oven was kept at 25°C with a flow rate of 0.3 ml/min. The mobile phase A consisted of water, 25 mM aqueous ammonia, and 25 mM ammonia, while phase B consisted of acetonitrile. Gradient elution conditions were set as follows: 0–0.5 min, 95% B; 0.5–7 min, linear from 95 to 65% B; 7–9 min, linear from 65 to 40% B; 9–10 min, 40% hold B; 10–11.1 min, linear from 40 to 95% B; 11.1–16 min, 95% hold B. The quality control samples were prepared to evaluate system stability.

### Mass spectrometric analysis of metabolites and data processing

The metabolites separated by chromatography were then analyzed using a mass spectrometer Triple-TOF 5600 (AB SCIEX, United States) with an electronic spray ionization (ESI) source operated in both positive and negative ion modes. The main parameter was set as following: ion source gas1/2: 60; curtain gas: 30; IonSpray voltage floating: ± 5,500 V; TOF MS m/z range: 60–1,200 Da; TOF scan time: 0.15 s/spectra; product ion scan m/z range: 25–1,200 Da; product ion scan time: 0.03 s/spectra. MS/MS spectra was obtained by information-dependent acquisition with a high sensitivity mode with the following parameters: declustering potential with ±60 V, collision energy of 30 eV, excluding isotopes within 4 Da, and candidate ions to monitor per cycle: 6.

After processing the raw data, the metabolites were identified *via* a self-built database (BioProfile Technology Co., Ltd., Shanghai, China) according to the accurate mass and MS/MS spectrogram. Furthermore, principal component analysis (PCA), partial least square discriminant analysis (PLS-DA), and orthogonal partial least squares discriminant analysis (OPLS-DA) were performed. Differentially accumulated metabolites (DAMs) were identified according to the following criteria: a variable influence on projection (VIP)> 1 and value of *p* < 0.05. Analysis on Gene Ontology (GO) and Kyoto Encyclopedia of Genes and Genomes (KEGG) pathway of DAMs was performed.

### DNA extraction, library construction and genome sequencing

Genomic DNA was extracted from the *Rhizobium* sp. WYJ-E13 using the CTAB method by Shanghai Personalbio Technology Co., Ltd. (Shanghai, China). The concentration of the DNA sample was evaluated using Quant-iT PicoGreen dsDNA Assay Kit, while the integrity was detected by 1% agarose gel. An insertion library of 400 bp pair-end was constructed with Illumina TruSeq DNA Sample Prep Kit according to manufacturer instructions and sequenced using an Illumina MiSeq instrument (Illumina, United States). Larger 20 k bp insertion (S20K) library was prepared by SMRTbell Template Prep Kit 1.0 according to the PacBio protocol, and sequenced using a PacBio Sequel I platform (Pacific Biosciences, United States).

### Genome assembly, annotation and gene prediction

The raw reads generated from Illumina and PacBio sequencing were utilized for bioinformatics investigation. For the Illumina short reads, the adaptor sequences were removed by Adapter Removal v2.1.7 (Schubert et al., 2016) and the k-mers for the paired-end libraries were tailed using SOAPec v2.0 (Luo et al., 2012). Furthermore, the sequencing reads were verified using SPAdes v3.9.0 (Bankevich et al., 2012), followed by assembly with A5-miseq v20150522 software (Tritt et al., 2017). Additionally, the PacBio sequencing data was assembled using HGAP4 (Chin et al., 2016) and CANU v1.6 (Koren et al., 2017). Subsequently, the assembled contigs from both Illumina and PacBio reads were merged, and gaps were filled. Finally, the full genome assembly was generated with Pilon v1.22 software (Walker et al., 2017).

GeneMarkS software v4.32 was used to predict protein-coding genes (Besemer et al., 2001). The protein-coding genes were aligned to the NCBI-NR database (Non-Redundant Protein Sequence Database) using diamond software v0.9.10.111 to retrieve annotations with an E-value cutoff of 1e-6 (Buchfink et al., 2015). Cluster of orthologous genes (COGs) was annotated with eggNOG-mapper with eggNOG database v4.5 and an E-value cutoff of 1e-6 (Huerta-Cepas et al., 2017). The KEGG orthology annotation was performed by KASS v2.1 software (Moriya et al., 2007). GO was annotated by InterPro v66.0 software (Finn et al., 2017). The annotation of Swiss-Prot was performed by BLAST with an E-value cutoff of 1e-6. tRNAscan-SE (Lowe and Eddy, 1997), Barrnap software (0.9-dev), and Rfam database (Burge et al., 2013) were used to predict tRNA, rRNA, and other non-coding RNAs, respectively. Clustered regularly interspaced short palindromic repeats (CRISPRs) finder tool was used to predict CRISPR regions (Grissa et al., 2007). The genome circular map of strain WYJ-E13 was drawn and visualized by CGView software (Stothard and Wishart, 2005).

### Comparative genome analysis

The average nucleotide identity (ANI) values among 16 genome sequences, including *Rhizobium* sp. WYJ-E13 and other 15 *Rhizobium* strains, were calculated using the JSpeciesWS online service (Richter et al., 2016). ANI results were used for hierarchical cluster analysis using TBtools v1.0987663 software.

Furthermore, collinearity of the conserved and highly orthologous genomic regions was analyzed using Mauve 20,150,226 software with default parameters (Darling et al., 2004). The locally collinear blocks (LCBs) showed the conserved and highly similar genomic region. The white areas inside colored regions indicated sequence elements specific to one genome that was not aligned. The height of similarity profile was present inside each block. The colored lines that connect LCBs represented translocations of homologous regions. Blocks above or below the horizontal bar indicated regions that aligned in the forward or reverse orientation, respectively.

## Results

### Isolation and characterization of endophytes from the root of *Curcuma wenyujin*

**Figure 1:**
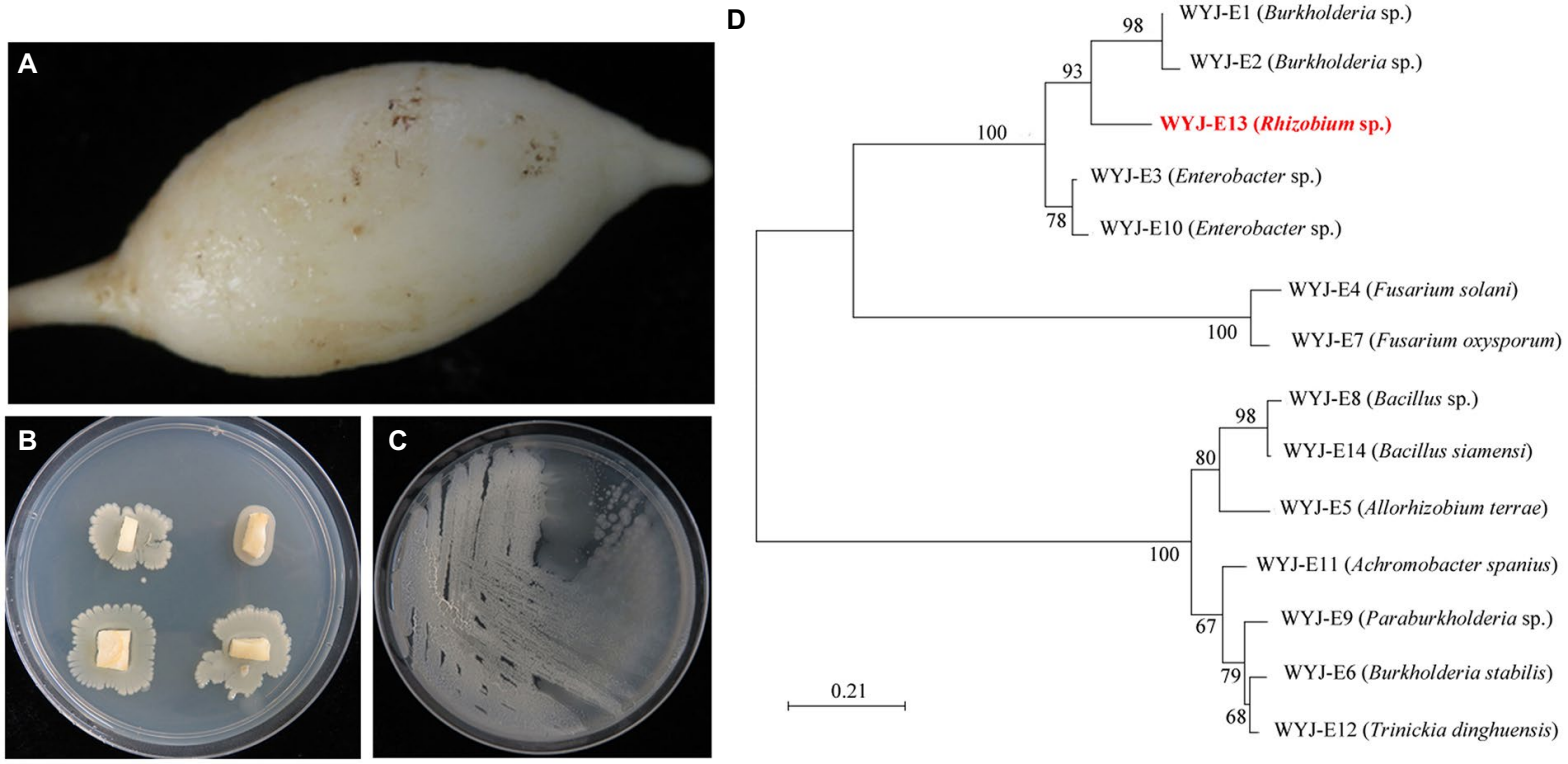
The isolation and characterization of endophytes isolated from the medicinal plant *Curcuma wenyujin*. **(A)** The tuberous root of medicinal plant *Curcuma wenyujin*. **(B)** The culture of endophytes on PDA medium. **(C)** The single-colony isolation of endophytes. **(D)** The phylogenetic tree of isolated 14 endophytes using MEGA X software.

In this study, the tuberous root of medicinal plant *C. wenyujin* was used as experimental materials (Figure 1A). After 3 days of growth on the PDA medium, different colonies spread from the cut surface of the tuberous root (Figure 1B). Single-colony isolation was repeated at least three times for purification of the endophyte isolates (Figure 1C). Based on the morphology, color, and growth characteristics, a total of 14 strains were isolated (Supplementary Figure S1).

Subsequently, the identification of all 14 strains was performed based on partial 16S rRNA gene sequencing. The obtained strain sequences were compared using the BlastN tool, with NCBI GenBank database nucleotide sequences and similarity values of ≥97% being obtained. The results showed the evolutionary relationships of all 14 strains (Figure 1D).

Considering the possible growth-promoting features, the *Rhizobium* sp. strain WYJ-E13 was selected for further research. Furthermore, the endophytic *Rhizobium* sp. strain WYJ-E13 was stored in the China General Microbiological Culture Collection Center at the Institute of Microbiology, Chinese Academy of Sciences, Beijing, China (strain preservation number CGMCC14808).

### Evaluation of plant growth promoting attributes

**Figure 2:**
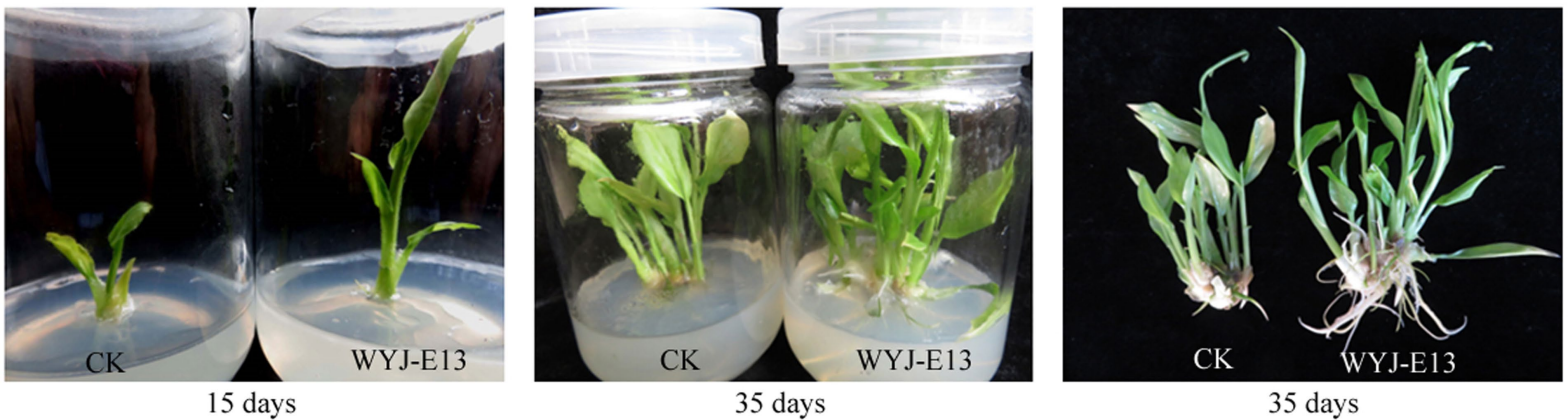
Plant growth promotion assay of *Rhizobium* sp. WYJ-E13 on the *Curcuma wenyujin* after 15 days and 35 days of inoculation.

For evaluating its potential of promoting plant growth, *Rhizobium* sp. strain WYJ-E13 was inoculated into the plantlets. The results showed a significant increase in growth rate, seedling height, and root length in the inoculated plantlets compared with not-inoculated control plants (*p* < 0.05), indicating that *Rhizobium* sp. strain WYJ-E13 could promote the growth of *C. wenyujin* (Figure 2).

### Colonization study of endophytic *Rhizobium* sp. strain WYJ-E13 on *Curcuma wenyujin*

**Figure 3:**
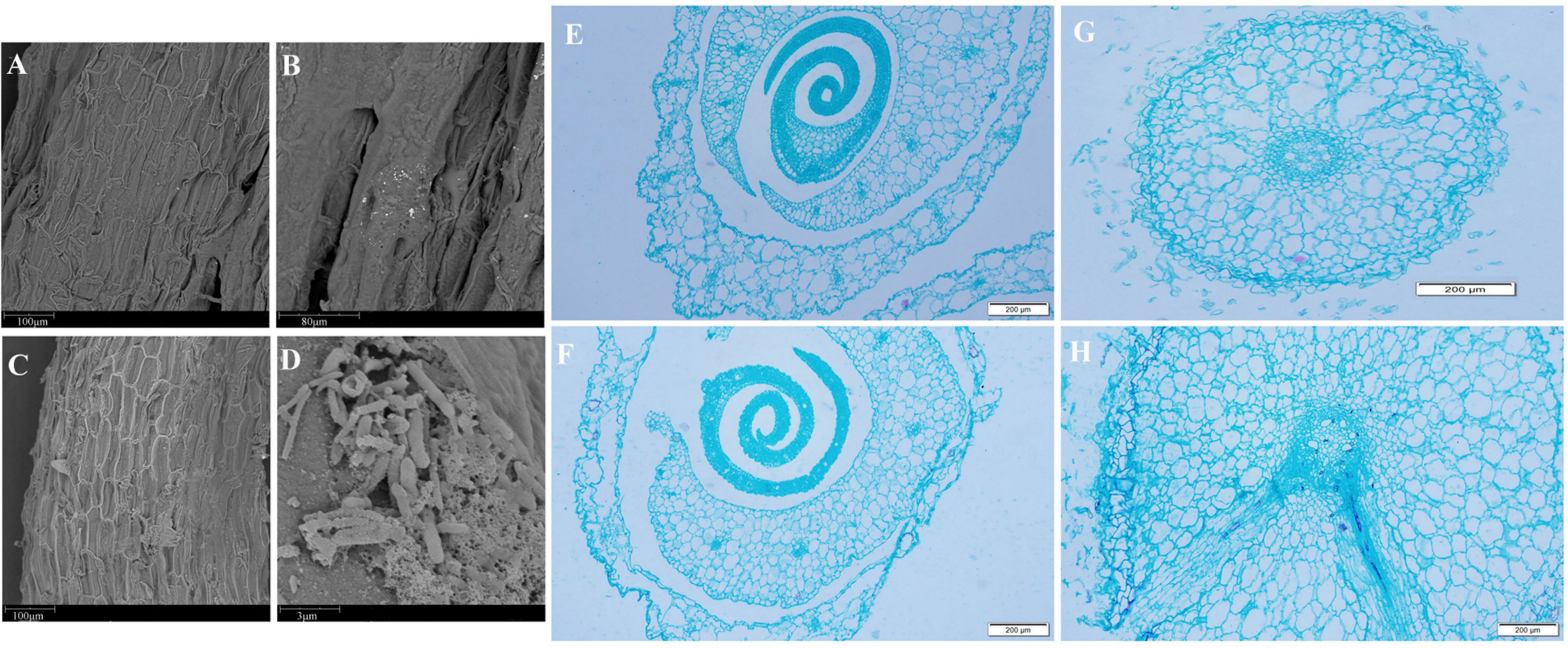
The observation of bacterium colonization by scanning electron microscopy (SEM) and paraffin section techniques. **(A,B)** The root colonization of *Curcuma wenyujin* without inoculation of WYJ-E13 by SEM. **(C,D)** The root colonization of *Curcuma wenyujin* after inoculation of WYJ-E13 by SEM. **(E)** The bud colonization without inoculation of WYJ-E13. **(F)** The bud colonization after inoculation of WYJ-E13. **(G)** The root colonization without inoculation of WYJ-E13. **(H)** The root colonization after inoculation of WYJ-E13.

The root colonization of *Rhizobium* sp. strain WYJ-E13 was examined by SEM, as this bacterium confirmed some plant growth promoting traits. These techniques helped study the interaction mechanism of the selected potential strains in *C. wenyujin*. After 35 days of incubation with the inoculated WYJ-E13 strain, SEM results confirmed the symbiotic colonization of WYJ-E13 in root tissues of *C. wenyujin* (Figures 3A–D). Furthermore, the morphology of endophytic *Rhizobium* sp. strain WYJ-E13 was rod-shaped (Figure 3D).

Besides, the paraffin sectioning of root and bud was observed by light microscope. The results showed that bacteria colonization was spotted in root tissues of *C. wenyujin* while no colonization was observed in the bud (Figures 3E–H), indicating the role of WYJ-E13 in root growth.

### Metabolites analysis of endophytic *Rhizobium* sp. strain WYJ-E13

In this study, the non-targeted metabolomics analysis was performed to explore possible metabolites secreted by the endophytic *Rhizobium* sp. strain WYJ-E13 that played potential promoting roles in plant growth. During the UPLC–MS/MS analysis process, the reproducibility of the instrument was monitored using the quality control (QC) samples, and the total ion chromatograms (TICs) of the QC samples in the positive (ESI +) and negative (ESI -) ions modes basically overlapped (Supplementary Figure S2). Furthermore, the PCA score plot of QC samples was tightly clustered near the origin (Supplementary Figure S3). These results indicated the stability of the instrument and the reliability of data generated from the LC–MS/MS analysis.

**Figure 4:**
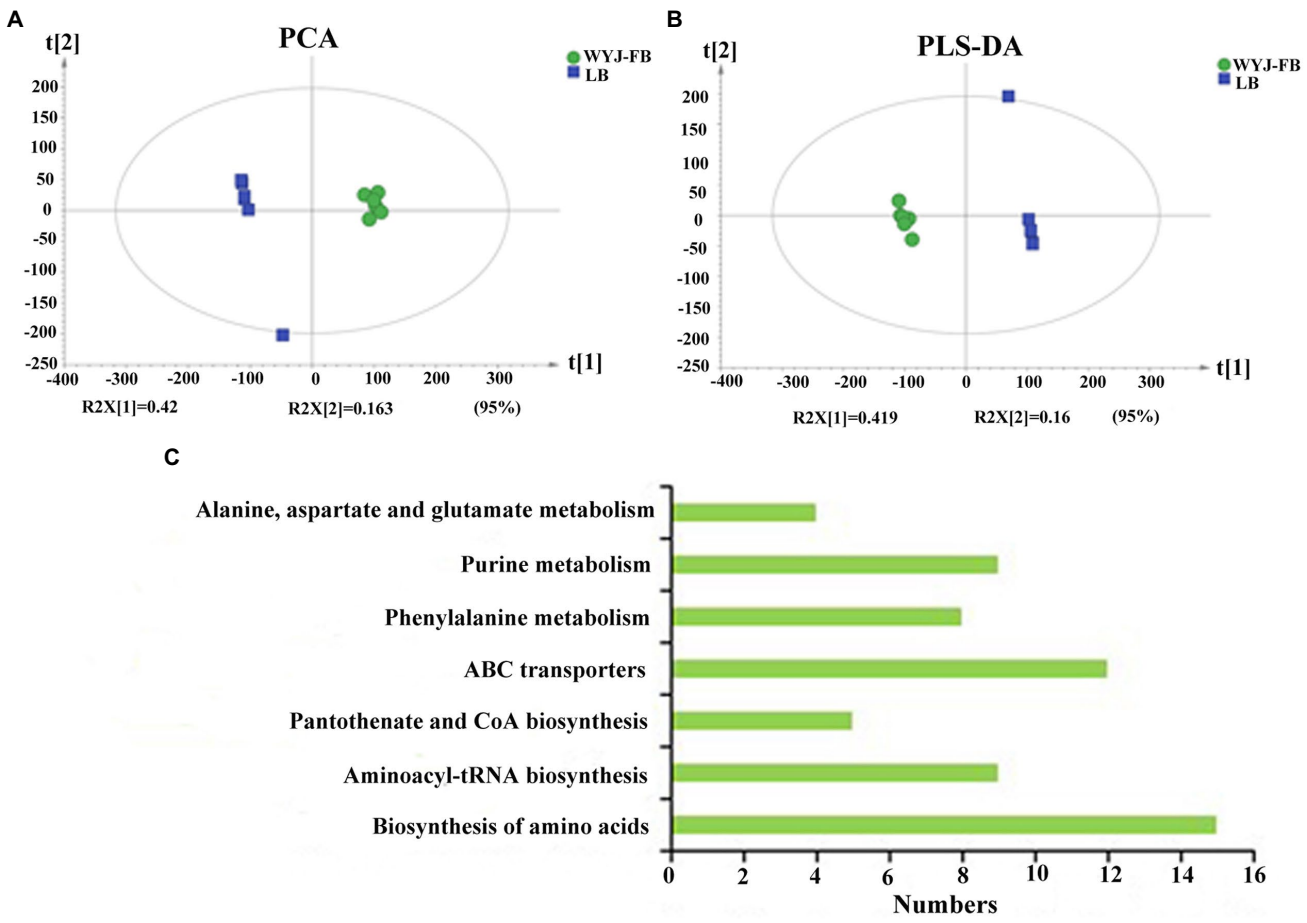
The metabolome analysis between WYJ-FB and LB medium. **(A)** PCA of the corrected and filtered data showed an obvious separation between WYJ-FB and LB medium. **(B)** The goodness-of-fit (R2) and predictability (Q2) of the PLS-DA models. **(C)** The KEGG pathway analysis of the identified differential metabolites produced by *Rhizobium* sp. WYJ-E13.

**Figure 5:**
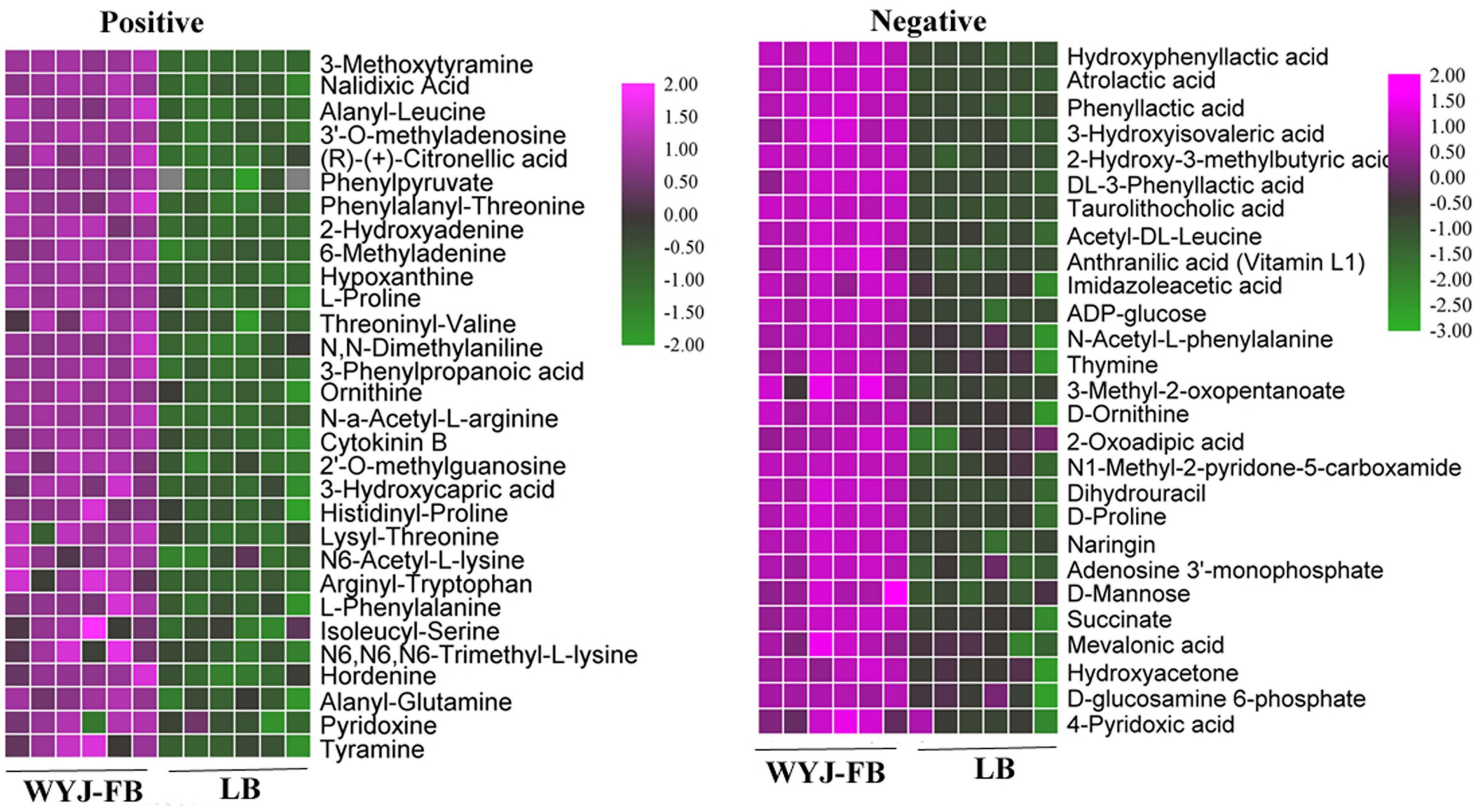
The heatmap of differential metabolites identified in positive ion modes and negative ion modes.

Using UPLC-MS/MS analysis, 13,283 positive ions and 13,303 negative ions were detected. PCA of the corrected and filtered data showed an obvious separation between WYJ-FB and LB medium (Figure 4A). The goodness-of-fit (R2) and predictability (Q2) of the PLS-DA models were higher than 0.95 in both WYJ-FB and LB medium (Figure 4B). In combination with the screening criterion of a VIP > 1.0, value of *p* < 0.05 and fold change (FC) > 2, a total of 1933 (ESI +) and 2,249 (ESI -) differential ions between WYJ-FB and LB medium were detected. Finally, 30 (ESI +) and 26 (ESI -) differential metabolites were obtained. Expectedly, the content of metabolites in WYJ-FB was higher than that in LB, regarding 3-methoxytyramine, nalidixic acid, phenylpyruvate, 6-methyladenine, hypoxanthine, cytokinin B and pyridoxine in positive ion modes (Table 1). In negative ion modes, the metabolites content of hydroxyphenyllactic acid, atrolactic acid, phenyllactic acid, 3-hydroxyisovaleric acid, 2-hydroxy-3-methylbutyric acid, and taurolithocholic acid were 100 folds higher than that in LB control medium (Table 2). Importantly, KEGG results showed that seven significantly enriched pathways were found, including biosynthesis of amino acids, aminoacyl-tRNA biosynthesis, pantothenate and CoA biosynthesis, ABC transporters, phenylalanine metabolism, purine metabolism and alanine, aspartate and glutamate metabolism (Figure 4C). Furthermore, the heatmap of differential metabolites was displayed (Figure 5). These results indicated that the *Rhizobium* sp. strain WYJ-E13 could produce various secondary metabolites.

**Table 1:** The differential metabolites identified in positive modes.

Metabolite (positive)	RT (s)	VIP	FC (FB/LB)	*p* value
3-Methoxytyramine	91.3515	1.43943	98.51257	2.13E-05
Nalidixic acid	632.4935	1.43783	54.5703	1.96E-05
Alanyl-leucine	71.406	1.22051	50.63086	0.002833
3’-O-methyladenosine	170.0675	1.52914	50.14315	2.36E-08
(R)-(+)-Citronellic acid	66.2015	1.24267	49.21178	0.002081
Phenylpyruvate	70.758	1.27098	47.74886	0.001018
Phenylalanyl-threonine	71.271	1.19395	35.11182	0.003985
2-hydroxyadenine	390.744	1.40403	33.98474	6.87E-05
6-methyladenine	210.448	1.44985	26.07884	1.45E-05
Hypoxanthine	275.975	1.54257	22.95381	1.87E-09
L-proline	884.821	1.50253	20.34587	5.13E-07
Threoninyl-valine	134.001	1.251	15.105	0.001839
N, N-dimethylaniline	72.072	1.30676	14.68333	0.000741
3-phenylpropanoic acid	91.2585	1.47128	12.93408	5.5E-06
Ornithine	884.861	1.50159	12.14966	4.94E-07
N-a-acetyl-L-arginine	272.604	1.5221	10.03841	8.79E-08
Cytokinin B	360.17	1.52255	9.281528	7.89E-08
2’-O-methylguanosine	335.042	1.44289	7.623098	1.65E-05
3-Hydroxycapric acid	220.653	1.32287	7.136805	0.000462
Histidinyl-Proline	361.625	1.3015	4.583888	0.000793
Lysyl-threonine	885.457	1.2765	3.792923	0.001214
N6-acetyl-L-lysine	148.855	1.32183	3.652186	0.000512
Arginyl-tryptophan	426.8495	1.25293	3.171163	0.001718
L-phenylalanine	362.298	1.39353	2.678859	9.16E-05
Isoleucyl-serine	132.37	1.00921	2.564513	0.023717
N6, N6, N6-Trimethyl-L-lysine	905.2335	1.16589	2.52011	0.004956
Hordenine	290.592	1.40078	2.467462	8.31E-05
Alanyl-glutamine	298.244	1.4538	2.343755	1.09E-05
Pyridoxine	187.7125	1.08029	2.310932	0.012376
Tyramine	359.393	1.29609	2.309834	0.000846

**Table 2:** The differential metabolites identified in negative modes.

Metabolite	RT (s)	VIP	FC (FB/LB)	*p* value
Hydroxyphenyllactic acid	417.079	1.41573	654.5715	4.31E-05
Atrolactic acid	211.036	1.47167	597.1147	4.44E-06
Phenyllactic acid	146.355	1.37144	482.5557	0.000174
3-Hydroxyisovaleric acid	332.332	1.13488	200.0901	0.007363
2-Hydroxy-3-methylbutyric acid	279.146	1.542	177.5169	2.64E-09
DL-3-Phenyllactic acid	212.888	1.37805	153.7545	0.000147
Taurolithocholic acid	475.9505	1.52309	119.4176	6.77E-08
Acetyl-DL-Leucine	395.6905	1.47628	25.45295	3.19E-06
Anthranilic acid	84.525	1.39078	17.57448	9.54E-05
Imidazoleacetic acid	88.507	1.39082	15.94654	1E-04
ADP-glucose	505.821	1.51595	12.41557	1.47E-07
N-acetyl-L-phenylalanine	365.7035	1.50294	10.15119	4.8E-07
Thymine	560.123	1.41984	8.327034	3.89E-05
3-methyl-2-oxopentanoate	80.358	1.20963	7.626435	0.002994
D-ornithine	882.994	1.47909	6.480002	2.86E-06
2-oxoadipic acid	621.363	1.38609	6.298379	0.000121
N1-methyl-2-pyridone-5-carboxamide	503.379	1.54424	6.252991	9.33E-10
Dihydrouracil	569.501	1.50372	6.14225	4.6E-07
D-proline	556.095	1.53429	4.540703	8.97E-09
Adenosine 3’-monophosphate	626.521	1.47105	3.109454	4.46E-06
D-mannose	736.5865	1.33633	2.890087	0.000408
Succinate	690.3615	1.47684	2.809224	3.36E-06
Mevalonic acid	373.3575	1.28582	2.399659	0.000978
Hydroxyacetone	488.552	1.40935	2.386334	6.02E-05
D-glucosamine 6-phosphate	699.345	1.39748	2.097681	8.52E-05
4-Pyridoxic acid	92.8665	1.00992	2.014605	0.023183

### Genome characteristics of *Rhizobium* sp. strain WYJ-E13

**Figure 6:**
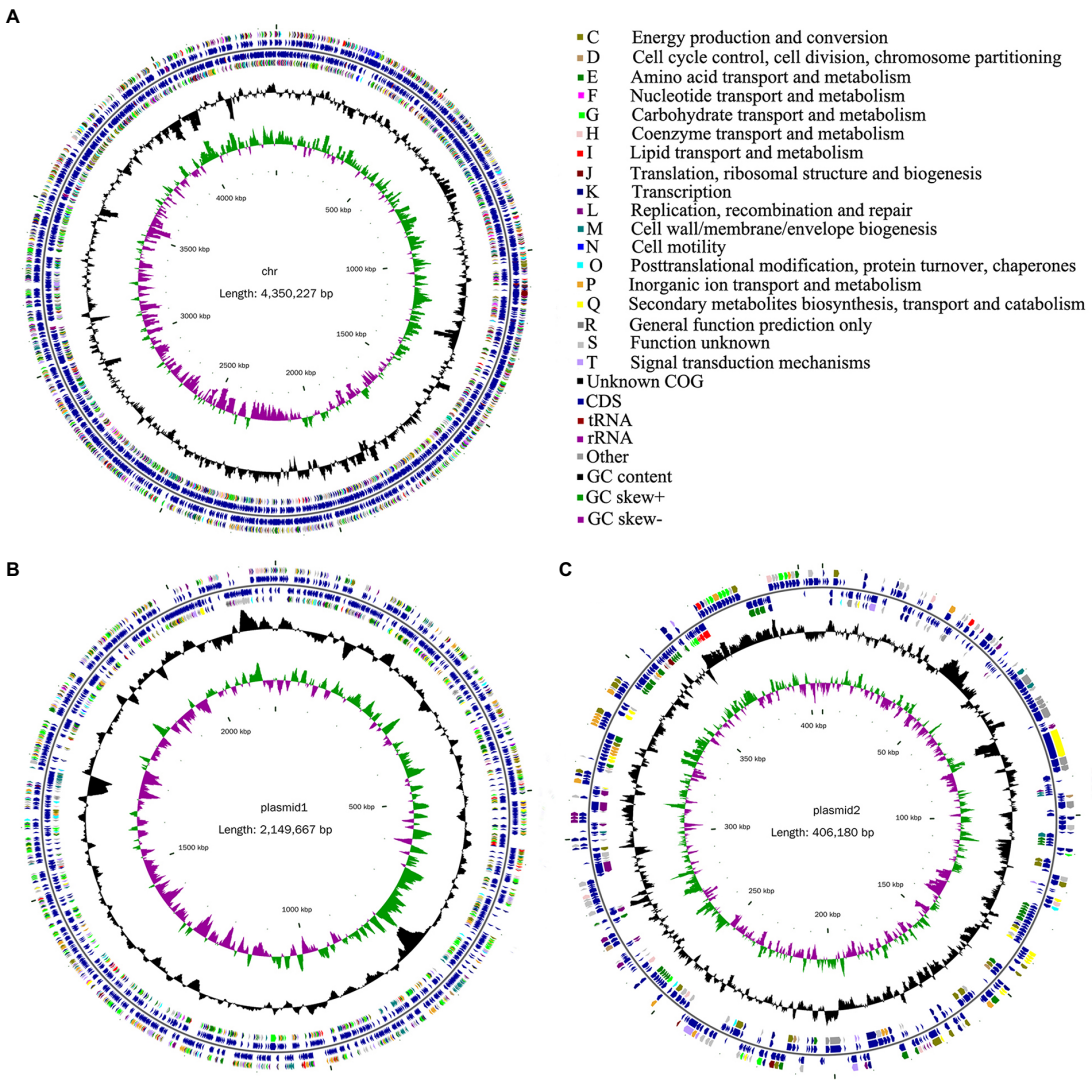
Circular representation of chromosome and plasmid of endophyte *Rhizobium* sp. WYJ-E13 strain isolated from the root of *Curcuma wenyujin*. **(A–C)** chromosome, plasmid1 and plasmid2, respectively; From the outer to the inner concentric circle: circle 1, genomic position in kilobases; circle 2, GC skew; circle 3, GC content; circle 4 and circle 7, the COG belonging to each CDS; circle 5 and circle 6, the position of CDS, tRNA and rRNA in the genome of strain WYJ-E13. Furthermore, C-T show the functional classification of the CDS genes in the chromosome and plasmid with the colors of the COG database.

In this study, a total of 3,741,172 raw reads were generated. After trimming, 3,663,822 high-quality reads were obtained on the Illumina MiSeq platform. The percentage of high-quality reads was 97.93%, reflecting the actual nucleotide composition. Furthermore, a total of 133,991 sequences were obtained on the PacBio Sequel I platform. The whole-genome characteristics of *Rhizobium* sp. strain WYJ-E13 were presented, which comprised of a circular chromosome of 4,350,227 bp with an overall GC content of 60.34%, of a 2,149,667 bp plasmid1 with 59.86% GC, and of a 406,180 bp plasmid2 with 58.05% GC (Table 3). The chromosome of WYJ-E13 displayed a clear GC skew transition, which corresponded to its replication origin and terminus (Figure 6A). There were 4,349 putative protein-coding genes, 51 tRNAs, and 9 rRNAs (Table 3). The characterization of predicted genes against KEGG, GO and COG database were 2027, 3,175 and 3,849 (Supplementary Figures S4–S6; Table 3). Furthermore, the plasmid1 genome annotations estimated protein coding with 2066 genes and plasmid2 with 435 protein coding genes (Figures 6B,C; Table 3).

**Table 3:** Genome characteristics of the *Rhizobium* sp. strain WYJ-E13.

Genome characteristics	Chromosome	Plasmid1	Plasmid2
Size(bp)	4,350,227	2,149,667	406,180
GC content	60.34%	59.86%	58.05%
Protein coding genes	4,349	2066	435
Genomic islands	184	2	3
CRISPR	3	0	0
tRNA	51	1	0
rRNA	9	0	0
Genes allocated to GO	3,175	1,582	251
Genes allocated to KEGG	2027	975	154
Genes allocated to COG	3,849	1792	307
Genes allocated to SwissProt	2,894	1,491	239
Genes allocated to NR	4,170	1933	360

CRISPRs are parts of prokaryotic DNA containing short base sequence repetitions, and CRISPR-related genes form a CRISPR-Cas system, which is an important defense system for organisms against foreign invaders. A total of three CRISPRs were also predicted from the chromosome sequence of WYJ-E13 with 81 bp shortest and 143 longest direct repeat sequences, no CRISPRs were found in both plasmid1 and plasmid2 (Table 3). Finally, a complete genome sequence of the *Rhizobium* sp. strain WYJ-E13 was submitted at GenBank with accession number CP076853-CP076855 (BioProject: PRJNA738292, BioSample: SAMN19717677).

### Comparative genome analysis

**Figure 7:**
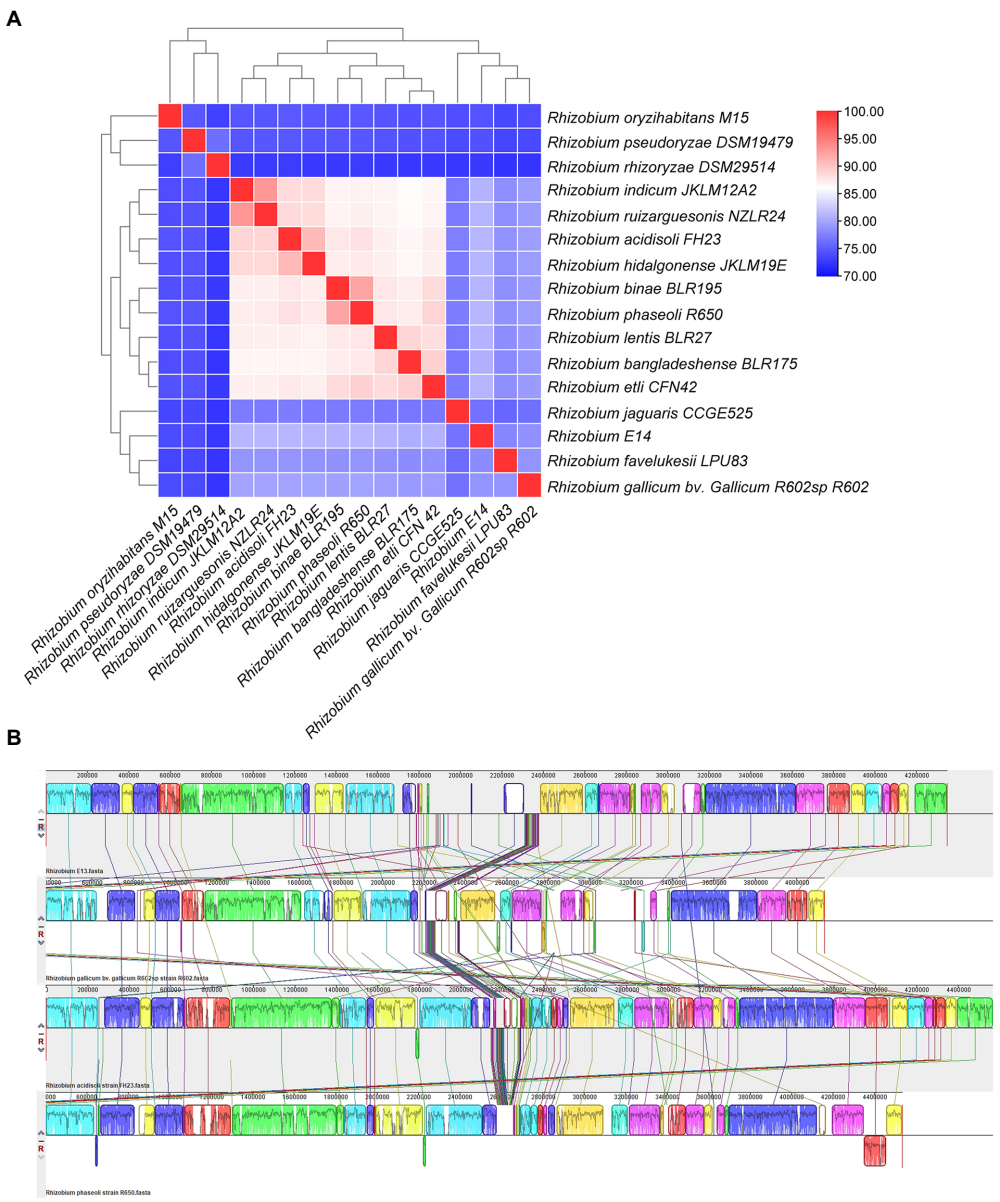
The average nucleotide identity analysis and comparative genome analysis. (**A**) The heat maps of ANI (average nucleotide identity) between strain WYJ-E13 and other 15 *Rhizobium* genus. (**B**) Genome-to-genome alignment of *Rhizobium* sp. WYJ-E13, *Rhizobium acidisoli* FH23, *Rhizobium gallicum* bv. *gallicum R602sp* strain R602 and *Rhizobium phaseoli* R650 used a progressive mauve software with a window of 1,000 nucleotides. WYJ-E13 was set as the reference genome. Boxes with the same color indicate the syntenic regions. Boxes below the horizontal line indicate inverted regions. Rearrangements are shown by colored lines.

The ANI value of *Rhizobium* sp. WYJ-E13 and other 15 strains were less than 95%, the highest value was 81.21% for *Rhizobium acidisoli* FH23 and *Rhizobium phaseoli* R650. The cluster analysis showed that *Rhizobium* sp. WYJ-E13 was a novel species (Figure 7A). Besides, three representative strains were selected for comparative genome analysis and the result of collinearity analysis was displayed (Figure 7B). In detail, 61.9% (7,633 genes), 40.8% (5,031 genes) and 60.7% (7,488 genes) of *Rhizobium* sp. WYJ-E13 have orthologous in the *R. acidisoli* FH23, *Rhizobium gallicum bv. gallicum R602sp* strain R602, and *R. phaseoli* R650, respectively.

### Genes associated with plant growth promoting in endophytic *Rhizobium* sp. WYJ-E13 genome

It has been reported that endophytes could promote the fitness and growth of host plants by secreting certain metabolites, such as nutrients and hormones. Thus, investigating the genes coded specific metabolites and involved in related biological processes in the genome of *Rhizobium* sp. WYJ-E13 is essential.

The annotation of the *Rhizobium* sp. strain WYJ-E13 genome identified 12 genes related to nitrogen metabolism including three nitrogen fixation genes (*chr_28*, *chr_35* and *chr_1434*), three glutamate synthase large subunit (*chr_2963*, *chr_3361* and *chr_3362*), four nitrate transporter (*chr_1,210*, *chr_1712*, *chr_2978* and *chr_3673*), a ferredoxin-nitrite reductase *chr_1392* and a glutamate dehydrogenase *chr_3941* (Table 4).

**Table 4:** Genes associated with plant growth promoting traits in *Rhizobium* sp. WYJ-E13 genome.

PGP activities	Gene ID	Gene name	Gene annotation	E.C. number	Chromosome location
Nitrogen metabolism	*chr_28*	*nifU*	Nitrogen fixation protein	/	25,045–25,611
	*chr_35*	*glnD*	Bifunctional nitrogen sensor protein	2.7.7.59	30,389–33,208
	*chr_1434*	*glnA*	Glutamine synthetase	6.3.1.2	1,413,233–1,414,642
	*chr_1,210*	*/*	ABC-type nitrate/sulfonate/bicarbonate transport system	/	1,192,777–1,193,604
	*chr_1392*	*/*	Sulfite reductase, beta subunit	/	1,364,037–1,365,707
	*chr_1712*	*/*	ABC transporter ATP-binding protein	/	1,698,945–1,699,775
	*chr_2963*	*/*	NADPH-dependent glutamate synthase beta chain	/	2,891,034–2,892,395
	*chr_2978*	*/*	ABC Transporter ATP-binding protein	/	2,906,472–2,907,257
	*chr_3361*	*gltD*	Glutamate synthase small subunit protein	/	3,313,120–3,314,667
	*chr_3362*	*gltB*	Glutamate synthase large subunit protein	/	3,314,800–3,319,524
	*chr_3673*	*/*	ABC-type nitrate/sulfonate/bicarbonate transport system	/	3,651,123–3,651,878
	*chr_3941*	*/*	Glutamate dehydrogenase	1.4.1.2	3,922,958–3,927,736
Hormone synthesis					
IAA production	*chr_31*	*trpS*	Tryptophan--tRNA ligase	6.1.1.2	26,579–27,643
	*chr_1483*	*trpD*	Anthranilate phosphoribosyltransferase	2.4.2.18	1,467,193–1,468,209
	*chr_1484*	*trpC*	Indole-3-glycerol phosphate synthase	4.1.1.48	1,468,219–1,469,031
	*chr_2801*	*trpE*	Anthranilate synthase	4.1.3.27	2,730,752–2,732,941
	*chr_3983*	*trpF*	anthranilate isomerase	5.3.1.24	3,971,953–3,972,594
	*chr_3984*	*trpB*	Tryptophan synthase beta chain	4.2.1.20	3,972,597–3,973,817
	*chr_3985*	*trpA*	Tryptophan synthase alpha chain	4.2.1.20	3,973,835–3,974,662
Cytokinin	*chr_1443*	*/*	cytokinin dehydrogenase activity	/	1,425,735–1,427,204
Jasmonic acid	*chr_1965*	*/*	methyl jasmonate esterase activity	/	1,946,243–1,947,553
Sulfur metabolism	*chr_574*	*cysN*	Sulfate adenylyltransferase subunit 1	2.7.7.4	584,482–585,966
	*chr_575*	*cysD*	Sulfate adenylyltransferase subunit 2	2.7.7.4	585,968–586,921
	*chr_1020*	*/*	OAH/OAS sulfhydrylase	/	1,007,839–1,009,122
	*chr_1193*	*/*	Cysteine synthase	/	1,178,261–1,179,301
	*chr_1335*	*cysE*	Serine O-acetyltransferase		1,307,445–1,308,278
	*chr_4316*	*cysK*	Cysteine synthase	2.5.1.47	4,315,608–4,316,570
Phosphorus metabolism	*chr_22*	*phoH*	Phosphate starvation-inducible protein	/	19,693–20,745
	*chr_149*	*pstC*	Phosphate ABC transporter permease subunit	/	146,756–148,204
	*chr_150*	*pstA*	Phosphate ABC transporter permease	/	148,201–149,523
	*chr_152*	*phoU*	Phosphate signaling complex protein	/	150,398–151,111
	*chr_153*	*phoB*	Phosphate regulon transcriptional regulator	/	151,243–151,926
	*chr_1036*	*/*	Phytase-like domain-containing protein	/	1,023,845–1,026,046
	*chr_1025*	*/*	Phosphate-binding protein PstS	/	1,011,224–1,012,246
Root colonization					
Motility	*chr_255*	*fliF*	Flagellar M-ring protein	/	254,515–256,143
	*chr_260*	*fliG*	Flagellar motor switch protein FliG	/	259,963–261,000
	*chr_261*	*FliN*	Flagellar motor switch protein FliN	/	261,029–261,655
	*chr_262*	*fliM*	Flagellar C-ring protein	/	261,722–262,669
	*chr_263*	*/*	Flagellar motor stator protein MotA	/	262,670–263,668
	*chr_264*	*flgF*	Flagellar basal-body rod protein FlgF	/	263,778–264,527
	*chr_265*	*/*	Flagellum-specific ATP synthase	7.1.2.2	264,542–265,942
	*chr_267*	*flgB*	Flagellar basal body rod protein FlgB	/	266,655–267,047
	*chr_268*	*flgC*	Flagellar basal-body rod protein FlgC	/	267,052–267,471
	*chr_269*	*fliE*	Flagellar hook-basal body complex protein FliE	/	267,471–267,812
	*chr_270*	*flgG*	Flagellar basal-body rod protein FlgG	/	267,832–268,620
	*chr_272*	*flgl*	Flagellar P-ring protein	/	269,116–270,237
	*chr_274*	*flgH*	Flagellar L-ring protein	/	270,764–271,477
	*chr_275*	*/*	Flagellar protein FliL	/	271,490–271,993
	*chr_278*	*flaCch1*	Flagellin	/	275,153–276,061
	*chr_279*	*flaCch2*	Flagellin	/	276,342–277,253
	*chr_285*	*FliK*	Flagellar hook-length control protein FliK	/	283,086–284,531
	*chr_288*	*FlgE*	Flagellar hook protein FlgE	/	286,117–287,403
Chemotaxis	*chr_248*	*cheA*	Histidine kinase	2.7.13.3	248,263–250,563
	*chr_251*	*cheB*	Protein-glutamate methylesterase/protein-glutamine glutaminase	3.1.1.61, 3.5.1.44	251,939–252,988
	*chr_253*	*cheD*	Probable chemoreceptor glutamine deamidase	3.5.1.44	253,374–253,928
	*chr_3307*	*/*	Histidine kinase	2.7.13.3	3,260,927–3,262,978
	*chr_3444*	*/*	Beta-glucosidase	3.2.1.21	3,411,475–3,412,848
	*chr_3565*	*lysC*	Aspartate kinase	2.7.2.4	3,547,497–3,548,771

Endophytes could enhance plant growth through the synthesis of hormone. In this study, seven IAA-related genes (*chr_31*, *chr_1483*, *chr_1484*, *chr_2801*, *chr_3983*, *chr_3984* and *chr_3985*), a cytokinin gene *chr_1443* and a jasmonic acid*-*related gene *chr_1965* were annotated in the genome of WYJ-E13 (Table 4).

Sulfur and phosphorus are vital nutrients for plant development. And a total of six sulfur assimilation-genes (*chr_574*, *chr_575*, *chr_1020*, *chr_1193*, *chr_1335* and *chr_4316*) and seven phosphorus-genes (*chr_22*, *chr_149*, *chr_150*, *chr_152*, *chr_153*, *chr_1036* and *chr_1025*) were annotated in the WYJ-E13 genome, respectively (Table 4).

Furthermore, the plant growth promoting attribute of WYJ-E13 firstly depends on its migration and colonization to the interior of host plants. Accordingly, the 18 motility-related genes and six chemotaxis-related genes were found in the WYJ-E13 genome. And additional information is presented in Table 4.

## Discussion

### Beneficial relationship between endophytic *Rhizobium* sp. WYJ-E13 to host plant *Curcuma*

It is well-known that the symbiotic fungi or bacteria that live asymptomatically within a healthy plant tissue are called endophytes (Trivedi et al., 2020). Nowadays, an increasing body of evidence points to the important roles that endophytes could play in the host plants. Thus, plant-endophyte interactions have gradually become a research hotspot, especially focusing on the benefits to their host.

Certainly, such a beneficial relationship could be displayed from three different aspects, including the promoting growth of their host plants, the secretion of bioactive compounds to increase the stress resistance of host plants, and the accumulation of important secondary metabolites produced by endophytes (Jia et al., 2016). Accordingly, mounting researches have paid attentions to the promoting effects of different endophytes on host plant growth, such as *P. variotii* (Moreno-Gavíra et al., 2020), *E. roggenkampii* ED5 (Guo et al., 2020), *P. aeruginosa* B18 (Singh, P., et al., 2021), *B. velezensis* LDO2 (Chen et al., 2019), *Microbacterium* spp. and *Streptomyces* spp. (Kataoka et al., 2021), *Rhizobium pusense* MB-17a (Chaudhary et al., 2021), *P. liquidambaris* (Zhou et al., 2022), *Bacillus altitudinis* (Zhang et al., 2021; Hasan et al., 2022), and *P. giganteum* (Yankey et al., 2022).

In this study, the inoculation experiment of *Rhizobium* sp. WYJ-E13 on the *C. wenyujin* was firstly performed and found that WYJ-E13 could promote the root growth of its host plant compared with the control (Figure 2). The result is consistent with the common perception that most of the rhizobial species are endophytes and colonize intracellularly for root growth promotion (Mus et al., 2016). Certainly, successful colonization is the prerequisite for endophytes to work (Khare et al., 2018; Harman et al., 2021). After inoculation of WYJ-E13 to its host plant *Curcuma*, the result of SEM observation confirmed the consensus (Figure 3D). To further explore which metabolites produced by endophytic *Rhizobium* sp. WYJ-E13 have growth-promoting effects and its encoding genes, the metabolome analysis and genome sequencing were performed. A total of 30 DAMs in positive ion modes were identified (Table 1) and 26 DAMs in negative ion modes (Table 2). Importantly, hormone cytokinin B was detected in metabolome (Table 1). Furthermore, the genome of *Rhizobium* sp. WYJ-E13 comprised of a chromosome and two plasmids (Figure 6 and Table 3). Moreover, a larger number of genes associated with nitrogen metabolism, hormone production, sulfur/phosphorus metabolism, motility and colonization were annotated (Table 4). These above-mentioned results may be responsible for the plant growth-promoting attributes of *Rhizobium* sp. WYJ-E13 to its host plant *C. wenyujin*. In addition, the growth promoting-related genes are discussed below in detail.

### Genes associated with nitrogen metabolism may play roles in growth of host plant *Curcuma*

Notoriously, the most efficient contribution to biologically fixed nitrogen is from symbioses between legumes and rhizobia (Pankievicz et al., 2019; Schulte et al., 2021). Recently, emerging evidence showed that some endophytes carried genes essential for biological nitrogen fixation to convert atmospheric N_2_ into ammonia and nitrate for secretion to the plant host (Ronson et al., 1981; Dubey et al., 2020; Mohr et al., 2021). For example, the endophytic *Stenotrophomonas* sp. and *Pseudomonas* sp. (Hazarika et al., 2021), *Pseudomonassp*.Y1 (Chen et al., 2022), *P. cypripedii* AF1 and *K. arachidis* EF1 (Singh, R. K., et al., 2021) are beneficial endophytes that can enhance the growth of their host plant by nitrogen fixation.

It has been reported that glutamate synthetases are the major enzymes of nitrogen metabolism (Liu et al., 2022). For example, Gene *nifU* is necessary for nitrogen fixation and takes part in the Fe-S cluster assembly (Smith et al., 2005). Gene *glnD* is essential for NifA activation, NtrB/NtrC-regulated gene expression, and posttranslational regulation of nitrogenase activity in the photosynthetic, nitrogen-fixing bacterium *Rhodospirillum rubrum* (Zhang et al., 2005). Deletion of type I glutamine synthetase (glnA) could deregulate nitrogen metabolism in *Clostridium thermocellum* (Rydzak et al., 2017). And the role of glutamate synthase gltBD in nitrogen metabolism of *Escherichia coli* has been demonstrated (Pahel et al., 1978; Goss et al., 2001). Interestingly, a total of 12 genes were annotated to be related to nitrogen metabolism, such as *nifU* (nitrogen fixation protein), *glnD* (bifunctional nitrogen sensor protein), *glnA* (glutamine synthetase), *gltD* (glutamate synthase small subunit protein) and *gltB* (glutamate synthase large subunit protein; Table 4). These genes were annotated in the WYJ-E13 genome, which further proved that the strain is directly connected with nitrogen metabolism.

### Genes associated with hormone may play roles in growth of host plant *Curcuma*

Endophytic bacterial strains promoted plant growth *via* diverse systems, such as producing IAA, cytokinin and jasmonic acid (Harman et al., 2021; Wu W. et al., 2021). For example, the plant growth-promoting ability of mycorrhizal *Fusarium* strain KB-3 was enhanced by its IAA producing endohyphal bacterium, *K. aerogenes* (Cheng et al., 2022). *Serratia marcescens* PLR enhanced lateral root formation through supplying PLR-derived auxin and enhancing auxin biosynthesis in Arabidopsis (Zhang et al., 2022). Certainly, the occurrence of tryptophan-linked genes was associated with IAA biosynthesis (Gupta et al., 2014; Asaf et al., 2018). For example, the tryptophan synthase β-subunit paralogs TrpB1 and TrpB2 in *Thermococcus kodakarensis* were both involved in tryptophan biosynthesis and indole salvage (Hiyama et al., 2014). And the genome of *P. aeruginosa* L10 also harbor tryptophan biosynthesis genes (t*rp ABCDEFG*) that were responsible for IAA synthesis (Wu et al., 2018). Interestingly, seven genes were annotated to be associated with IAA production in the genome of WYJ-E13, such as *trpB* (tryptophan synthase beta chain), *trpC* (indole-3-glycerol phosphate synthase), and *trpD* (anthranilate phosphoribosyltransferase; Table 4).

Besides, cytokinins also play a significant role in plant growth promotion (Gao et al., 2022). For example, cytokinin production by *Azospirillum brasilense* contributed to increase in growth, yield, antioxidant, and physiological systems of wheat (Zaheer et al., 2022). Expectedly, a gene *chr_1443* was annotated to possess cytokinin dehydrogenase activity (Table 4). In addition, *Bacillus circulans* GN03 altered the microbiota, and promoted cotton seedling growth by increasing the expression of phytohormone jasmonic acid synthesis (Qin et al., 2021). Similarly, a gene *chr_1965* was annotated to have methyl jasmonate esterase activity (Table 4). The presence of hormone-associated genes may reveal the possible reason why WYJ-E13 promoted plant growth.

### Genes associated with sulfur/phosphorus metabolism may play roles in growth of host plant *Curcuma*

Sulfur is one of the essential nutrients that is required for the adequate growth and development of plants (Narayan et al., 2022). For example, the phenotype of multiple Arabidopsis sulfur metabolism mutants was partially or completely rescued by the plant growth-promoting bacteria *Enterobacter* sp. SA187 as much as by the addition of sulfate, implying its regulation of sulfur metabolic pathways (Andrés-Barrao et al., 2021). The combined treatment of JIL321 and NaHS could further improve the growth of rice seedlings, most likely due to the interaction effect between H_2_S and strain JIL321 (Wang et al., 2022). In addition, the *cysP* gene was functionally verified with a strain *E. coli* transformed by expressing *B. subtilis cysP* gene through a mutated sulfate transport (Mansilla and de Mendoza, 2000). The operon determined by *cysP* gene in *B. subtilis* was accountable for sulfur metabolism, for example, the sulfate adenylyltransferase gene (Aguilar-Barajas et al., 2011). Coincidently, two genes *cysD* and *cysN* (sulfate adenylyltransferase subunit) were found to be annotated in the genome of WYJ-E13 (Table 4).

Furthermore, phosphorus was another vital and limiting macronutrient for plant growth. Specific bacteria played an important role in supplying accessible inorganic phosphorous in the form of orthophosphate to the plant, owing to phosphate generally existing in the soil in the form of insoluble compounds and plants are only proficient to receive free orthophosphate (Bergkemper et al., 2016). A highly efficient phosphorus solubilizing bacteria strain *Pseudomonas* sp. JP233 exhibited a significant plant growth-promoting effect on maize development (Yu et al., 2022). And phosphorus-related genes (*pstA*, *pstC*, *phoB*, *phoH*, *phoU*) were annotated in the genome of WYJ-E13 (Table 4). Moreover, phytase-producing bacteria were also proved to promote plant growth (Singh et al., 2014). Interestingly, a gene chr_1036 was annotated as phytase-like domain containing protein in the genome of WYJ-E13 (Table 4), which may further imply the possible roles of WYJ-E13 in promoting growth of host plant.

### Genes associated with root colonization may play roles in growth of host plant *Curcuma*

Generally, beneficial microorganism colonized on the tissues of various sections in the plant. The interactions between beneficial microbes of the host plant might play an essential part in the achievement of microbial bioinoculant for promoting the growth of plant. The SEM images confirmed the colonization of *Rhizobium* sp. WYJ-E13 in the root of *C. wenyujin* (Figure 3). Furthermore, motility and chemotaxis were important characteristics for endophytes (Taghavi et al., 2010). Recently, the biofilm formation of diazotrophic bacteria *Gluconacetobacter diazotrophicus* was associated with the stimulation of certain compounds, and promoted its motility and colonization into the host plant, thus further improving biological nitrogen fixation with increased grain yield (Yan et al., 2022). In WYJ-E13 genome, a total of 18 genes were associated with motility (Table 4).

Besides, about half of the sequenced bacterial genomes contained genes encoding chemotactic signaling cascades (Sanchis-López et al., 2021). One of the benefits of chemotaxis accessed to nutrients and promoted its host colonization (Corral-Lugo et al., 2016). For example, the *B. velezensis* SQR9 was a commercially widely used plant growth-promoting rhizobacterium, its dCache LBD containing chemoreceptor McpA plays a critical role in chemotaxis to root exudates, biofilm formation, and rhizosphere colonization (Shao et al., 2015; Feng et al., 2019). Similarly, six genes were annotated to be associated with chemotaxis in the genome of WYJ-E13 (Table 4), which implied the roles of root colonization in plant growth promotion.

## Conclusion

This is the first report of experimental confirmations of the endophyte *Rhizobium* sp. strain WYJ-E13 as a PGPR isolated from the root of medicinal plant *C. wenyujin*. The bacterium exhibited plant growth-promoting activities for the enhanced root growth of *C. wenyujin*. Additionally, hormone cytokinin produced by the strain WYJ-E13 might be one of the reasons in growth-promotion. Most important, the WYJ-E13 genome carried a total set of genes associated with nitrogen metabolism, hormone synthesis, sulfur/phosphorus metabolism and root colonization. So, it can be summarized that the WYJ-E13 strain may be used as a possible alternate for biofertilizer and play a role in growth promotion. However, field trials are required to explain the usability of the *Rhizobium* sp.WYJ-E13 in the field earlier than it can be established as a plant growth promoter for utilizing in agriculture.

## Data availability statement

The datasets presented in this study can be found in online repositories. The names of the repository/repositories and accession number(s) can be found at: https://www.ncbi.nlm.nih.gov/genbank/, CP076853; https://www.ncbi.nlm.nih.gov/genbank/, CP076854; https://www.ncbi.nlm.nih.gov/genbank/, CP076855.

## Author contributions

XH drafted the manuscript. ZZ and ZC provided the bioinformatics analysis. XT isolated the endophytes. JJ and CH provided the assistance in experiments. XH and TX designed the experiment, and provided the overall guidance and revision. All authors contributed to the article and approved the submitted version.

## Funding

The work was supported by grants from the National Natural Science Foundation of China (grant no. 31872181) and Hangzhou Science and Technology Development Plan (grant no. 20191203B05 and 20211231Y088).

## Conflict of interest

The authors declare that the research was conducted in the absence of any commercial or financial relationships that could be construed as a potential conflict of interest.

## Publisher’s note

All claims expressed in this article are solely those of the authors and do not necessarily represent those of their affiliated organizations, or those of the publisher, the editors and the reviewers. Any product that may be evaluated in this article, or claim that may be made by its manufacturer, is not guaranteed or endorsed by the publisher.

## Supplementary material

Supplementary Figure S1 The 14 endophytic strains isolated from the root of medicinal plant *Curcuma wenyujin*.

Supplementary Figure S2 The total ion chromatograms (TICs) of the QC samples in the positive (ESI +) and negative (ESI -) ions modes.

Supplementary Figure S3 The PCA score plot of QC samples.

Supplementary Figure S4 The characterization of predicted genes against KEGG database.

Supplementary Figure S5 The characterization of predicted genes against GO database.

Supplementary Figure S6 The characterization of predicted genes against COG database.
