# Supplementary figures and images for: Deciphering the potential of a plant growth promoting endophyte Rhizobium sp. WYJ-E13, and functional annotation of the genes involved in the metabolic pathway
Source: Front Microbiol. 2022 Nov 3;13:1035167. doi: 10.3389/fmicb.2022.1035167 (PMC9671153; doi:10.3389/fmicb.2022.1035167)

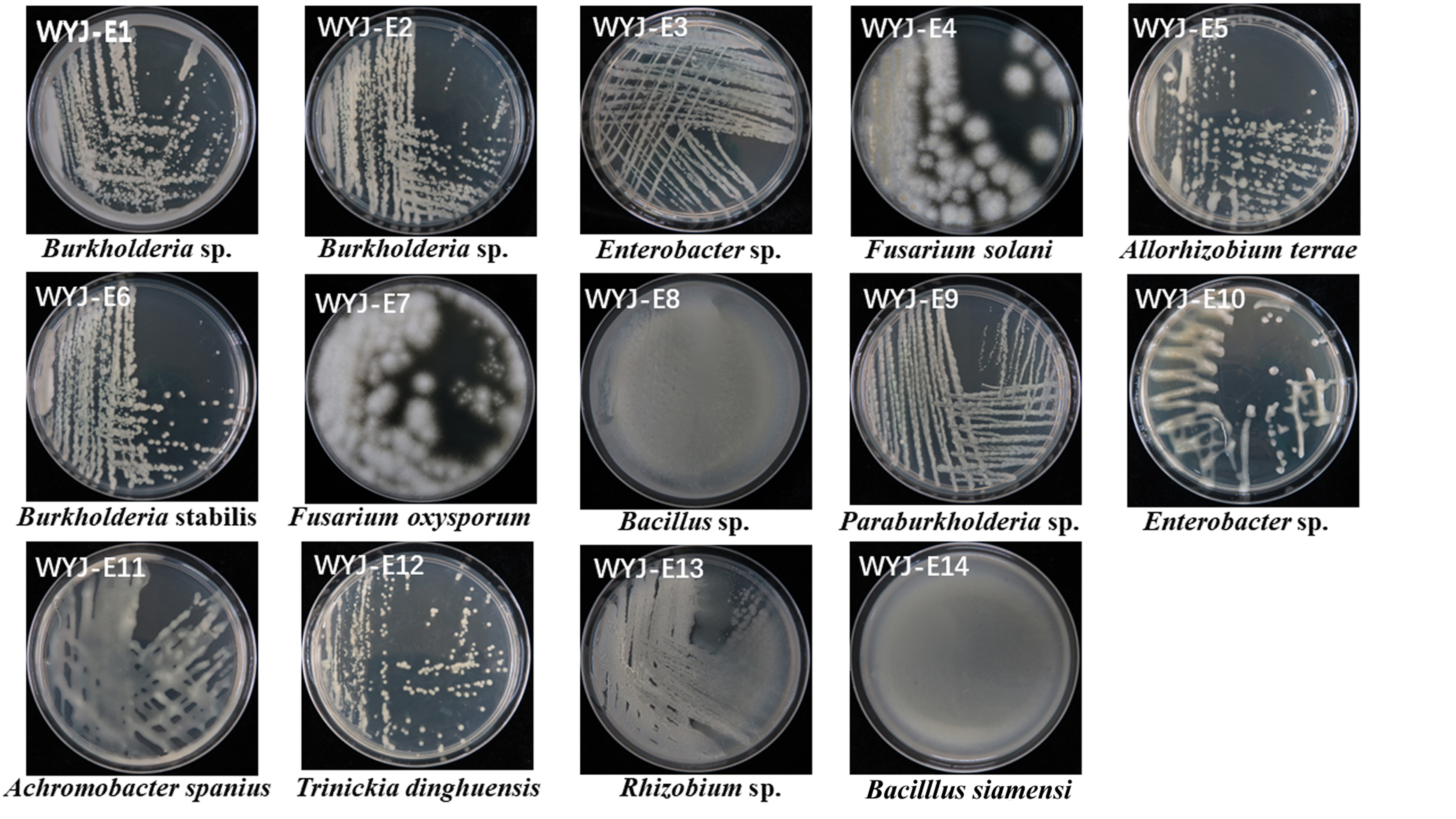

Supplement: Supplementary Figure S1 — The 14 endophytic strains isolated from the root of medicinal plant Curcuma wenyujin. [file Image_1.JPEG]

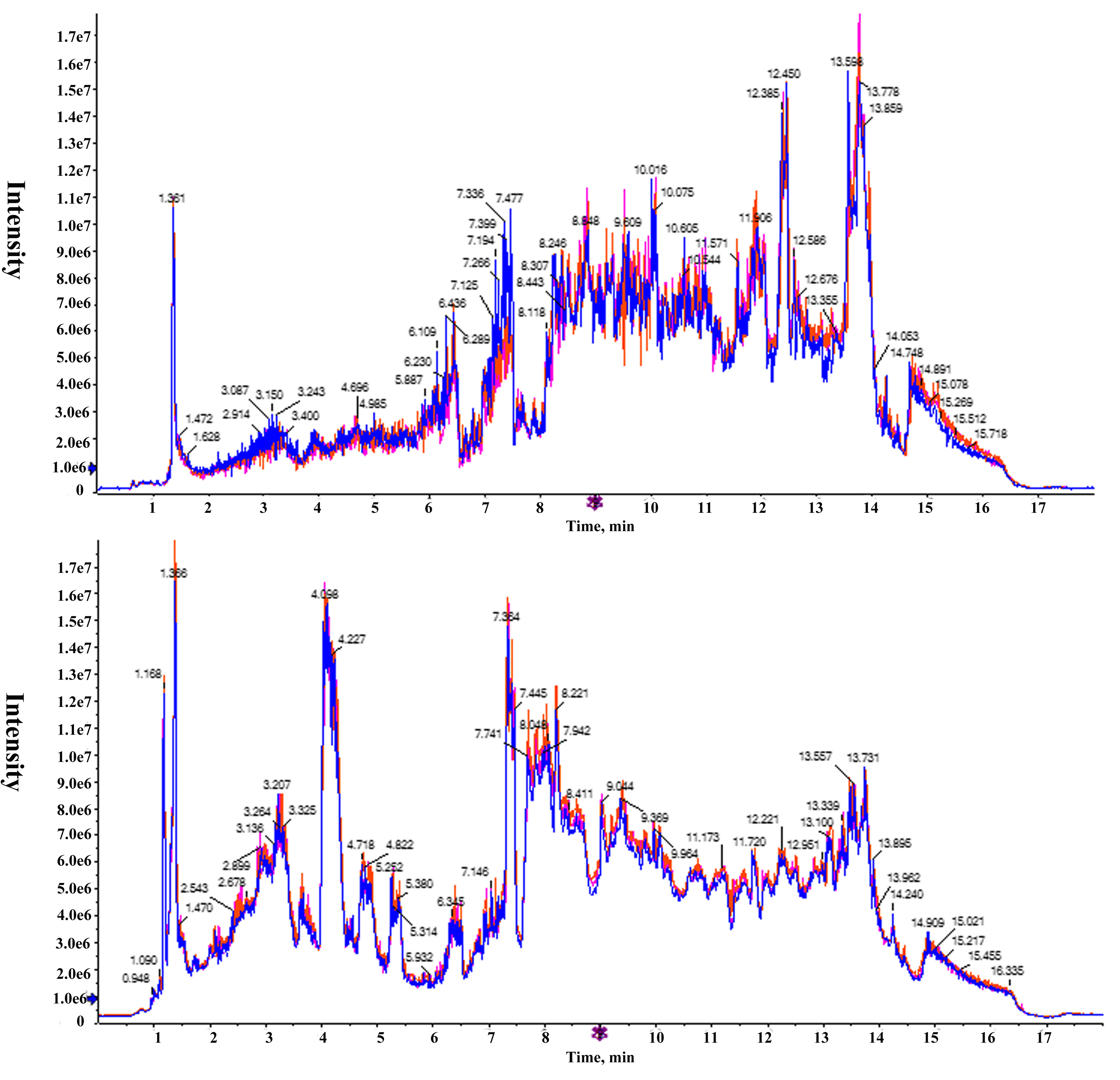

Supplement: Supplementary Figure S2 — The total ion chromatograms (TICs) of the QC samples in the positive (ESI +) and negative (ESI -) ions modes. [file Image_2.JPEG]

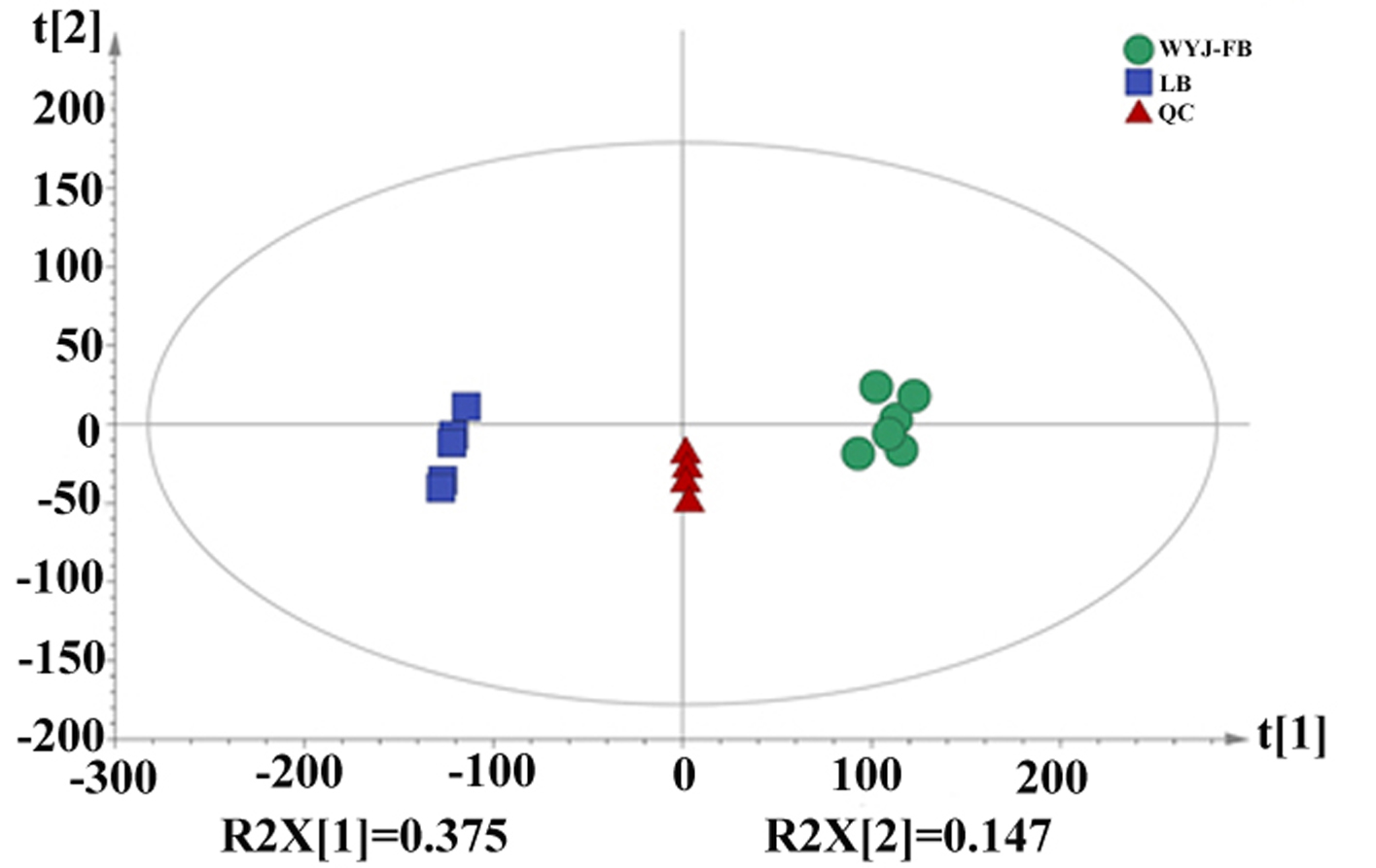

Supplement: Supplementary Figure S3 — The PCA score plot of QC samples. [file Image_3.JPEG]

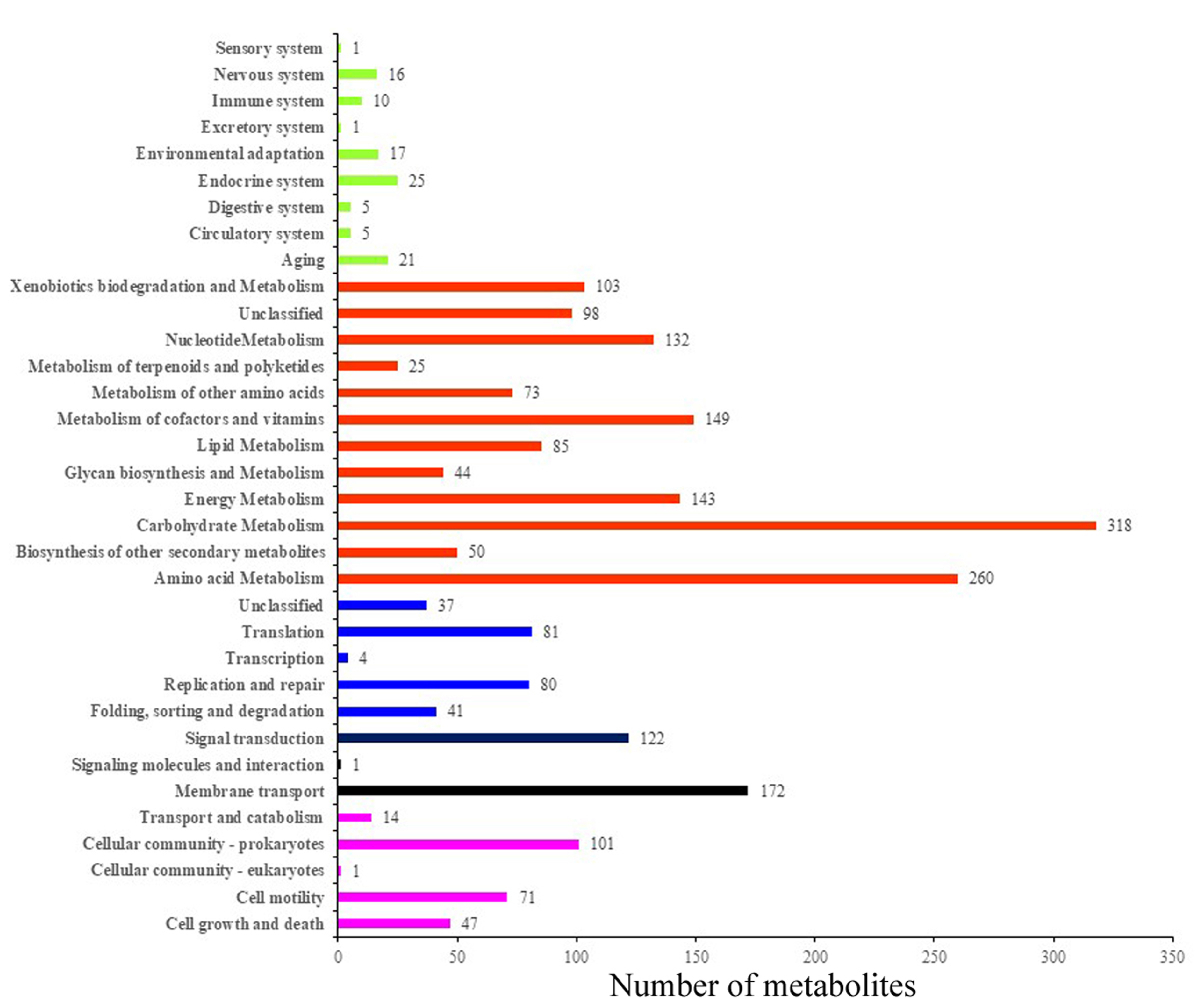

Supplement: Supplementary Figure S4 — The characterization of predicted genes against KEGG database. [file Image_4.JPEG]

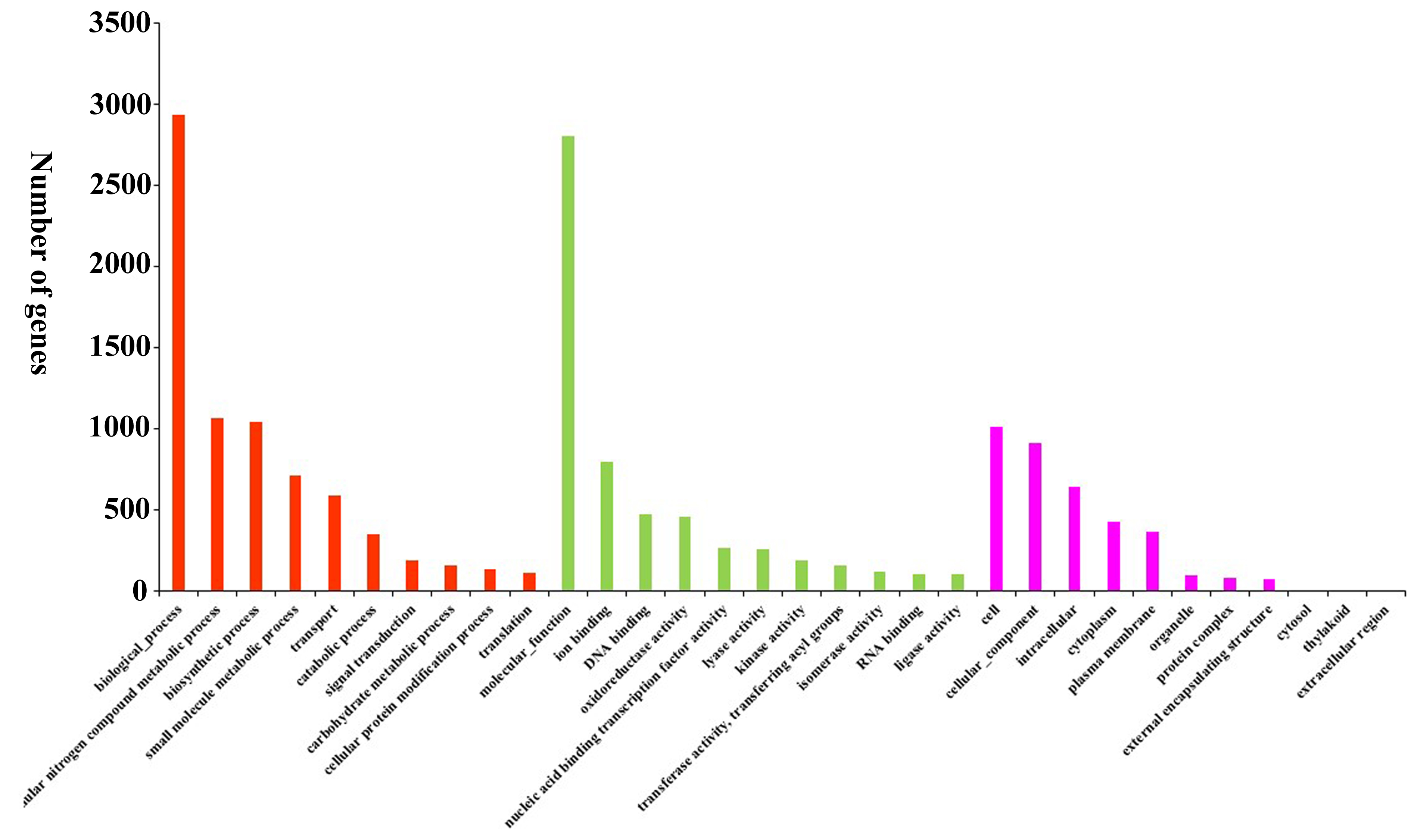

Supplement: Supplementary Figure S5 — The characterization of predicted genes against GO database. [file Image_5.JPEG]

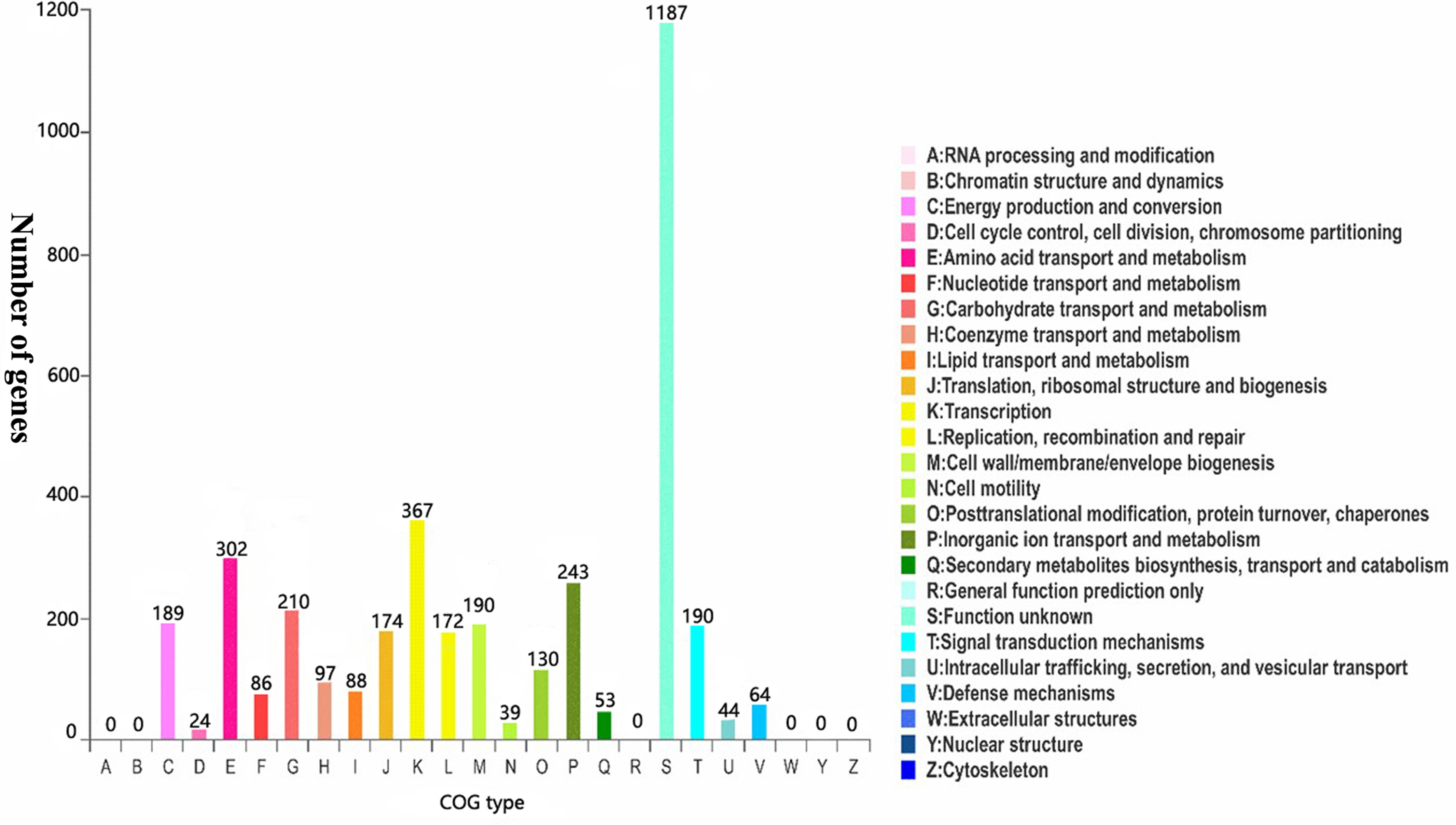

Supplement: Supplementary Figure S6 — The characterization of predicted genes against COG database. [file Image_6.JPEG]
